# Articulatory speech measures can be related to the severity of multiple sclerosis

**DOI:** 10.3389/fneur.2023.1075736

**Published:** 2023-06-13

**Authors:** Maiara Laís Mallmann Kieling, Alessandro Finkelsztejn, Viviana Regina Konzen, Vanessa Brzoskowski dos Santos, Annelise Ayres, Iasmin Klein, Rui Rothe-Neves, Maira Rozenfeld Olchik

**Affiliations:** ^1^Post-Graduate Program in Medicine, Medical Sciences, Universidade Federal do Rio Grande do Sul, Porto Alegre, RS, Brazil; ^2^Neurology Service, Hospital de Clínicas de Porto Alegre, Porto Alegre, RS, Brazil; ^3^Speech Language Pathology Course, Universidade Federal do Rio Grande do Sul, Porto Alegre, RS, Brazil; ^4^Phonetics Laboratory of the Faculty of Letters, Universidade Federal de Minas Gerais, Belo Horizonte, MG, Brazil; ^5^Department of Surgery and Orthopedics, Faculdade de Odontologia, Universidade Federal do Rio Grande do Sul, Porto Alegre, RS, Brazil

**Keywords:** dysarthria, speech disorder, multiple sclerosis, speech therapy assessment, speech acoustics

## Abstract

**Background:**

Dysarthria is one of the most frequent communication disorders in patients with Multiple Sclerosis (MS), with an estimated prevalence of around 50%. However, it is unclear if there is a relationship between dysarthria and the severity or duration of the disease.

**Objective:**

Describe the speech pattern in MS, correlate with clinical data, and compare with controls.

**Methods:**

A group of MS patients (*n* = 73) matched to healthy controls (*n* = 37) by sex and age. Individuals with neurological and/or systemic conditions that could interfere with speech were excluded. MS group clinical data were obtained through the analysis of medical records. The speech assessment consisted of auditory-perceptual and speech acoustic analysis, from recording the following speech tasks: phonation and breathing (sustained vowel/a/); prosody (sentences with different intonation patterns) and articulation (diadochokinesis; spontaneous speech; diphthong/iu/repeatedly).

**Results:**

In MS, 72.6% of the individuals presented mild dysarthria, with alterations in speech subsystems: phonation, breathing, resonance, and articulation. In the acoustic analysis, individuals with MS were significantly worse than the control group (CG) in the variables: standard deviation of the fundamental frequency (*p* = 0.001) and maximum phonation time (*p* = 0.041). In diadochokinesis, individuals with MS had a lower number of syllables, duration, and phonation time, but larger pauses per seconds, and in spontaneous speech, a high number of pauses were evidenced as compared to CG. Correlations were found between phonation time in spontaneous speech and the Expanded Disability Status Scale (EDSS) (*r* = − 0.238, *p* = 0.043) and phonation ratio in spontaneous speech and EDSS (*r* = −0.265, *p* = 0.023), which indicates a correlation between the number of pauses during spontaneous speech and the severity of the disease.

**Conclusion:**

The speech profile in MS patients was mild dysarthria, with a decline in the phonatory, respiratory, resonant, and articulatory subsystems of speech, respectively, in order of prevalence. The increased number of pauses during speech and lower rates of phonation ratio can reflect the severity of MS.

## 1. Introduction

Multiple Sclerosis (MS) is the most common autoimmune, demyelinating, and chronic disease of the central nervous system (CNS) in young adults (1). MS comes in three different subtypes: relapsing–remitting (RRMS), secondary-progressive (SPMS), and primary-progressive (PPMS) (2). The disease manifests with different clinical phenotypes depending on the site affected by the demyelinating lesions in the central nervous system (1, 2). These patients may present neurological, cognitive, physical, and motor symptoms, such as limb weakness, gaits, fatigue, ataxia, disturbances of sensitivity and visual changes. Relapses, also known as “flare-ups,” can start from insidious or abrupt forms, varyingly, from mild symptoms such as limiting changes. Young individuals are most often affected, especially women between 20 and 40 years old (3). RRMS is the most common and mildest manifestation of the disease. It is estimated that 85% of patients have MS in this form (4). Although, RRMS can also manifest with severe clinical symptomatology. SPMS is an evolution of untreated RRMS cases or patients that present constant changes in the disease symptoms. The PPMS, on the other hand, shows a progression of symptoms and sequelae since its onset, the most aggressive form of the disease since the patient is constantly active in demyelination (5).

Changes in the communication of individuals with MS are common (6), with dysarthria being one of the most frequent symptoms, with a prevalence between 46–56% (7–9). Dysarthria results from alterations in motor processing in speech, which involve five subsystems: breathing, phonation, resonance, articulation, and prosody (10–12). Self-reported questionnaires related to dysarthria indicate prevalence ranging from 23 to 56% (8). Different alterations in speech subsystems in patients with MS have already been described, and the most common are increased number of pauses, slowed articulation, altered intensity control, monopitch, articulatory imprecision of consonants, and decreased respiratory capacity (13, 14). Cognitive impairment may also have an influence on motor speech alterations (15).

Most studies show a higher incidence of mild dysarthria in patients with MS, with severe manifestations found in patients with advanced neurological conditions (13, 14, 16). The progression of dysarthria parallels the advance of the neurological panorama of the disease, although this relationship is inconclusive. Therefore, this study aims to characterize the speech pattern of patients with MS through auditory-perceptual and acoustic speech analysis, correlate with their clinical characteristics and compare with controls.

## 2. Materials and methods

Cross-sectional descriptive study.

### 2.1. Participants

Patients diagnosed with MS from the Neuroimmunology Outpatient Clinic of Hospital de Clínicas de Porto Alegre (HCPA), Brazil, were included based on the 2017 revision of the McDonald diagnostic criteria (17). Recruitment took place consecutively in the clinic. Exclusion criteria were other associated neurological or systemic diseases that could interfere with speech, MS relapse in the last three months, and severe hearing loss. Healthy people not associated with the patients were recruited from the local community to form the control group, matched by sex and age. To rule out any disease that could interfere with the speech in the control group, all individuals answered questions about diagnoses, surgeries, and medication use. The exclusion criteria were history of other previous neurological events, sensory or motor disorders, systemic diseases and/or structural changes that affected the voice and/or speech. All participants had Brazilian Portuguese as their native language. The HCPA Ethics Committee approved the study under Nr. GPPG 2019-0789, and all participants signed informed consent.

### 2.2. Clinical and sociodemographic data

Clinical and sociodemographic data were collected from the patient’s electronic medical record at the medical consultation performed on the same day as the speech assessment. The variables collected were age, sex, disease history, the age of onset of symptoms, diagnosis time, smoking, and the patient’s current neurological status, Expanded Disability Status Scale (EDSS) (18) score. The EDSS is currently the most frequently used scale to evaluate MS disability. It describes disease progression in patients with MS and assesses the effectiveness of therapeutic interventions. The scale evaluates the eight functional systems monitoring pyramidal, cerebellar, brainstem, sensory, bowel and bladder, visual, mental, and other functions. It consists of an ordinal rating system ranging from 0 (normal neurological status) to 10 (death due to MS) in 0.5 increments intervals (when reaching EDSS 1).

**Figure 1:**
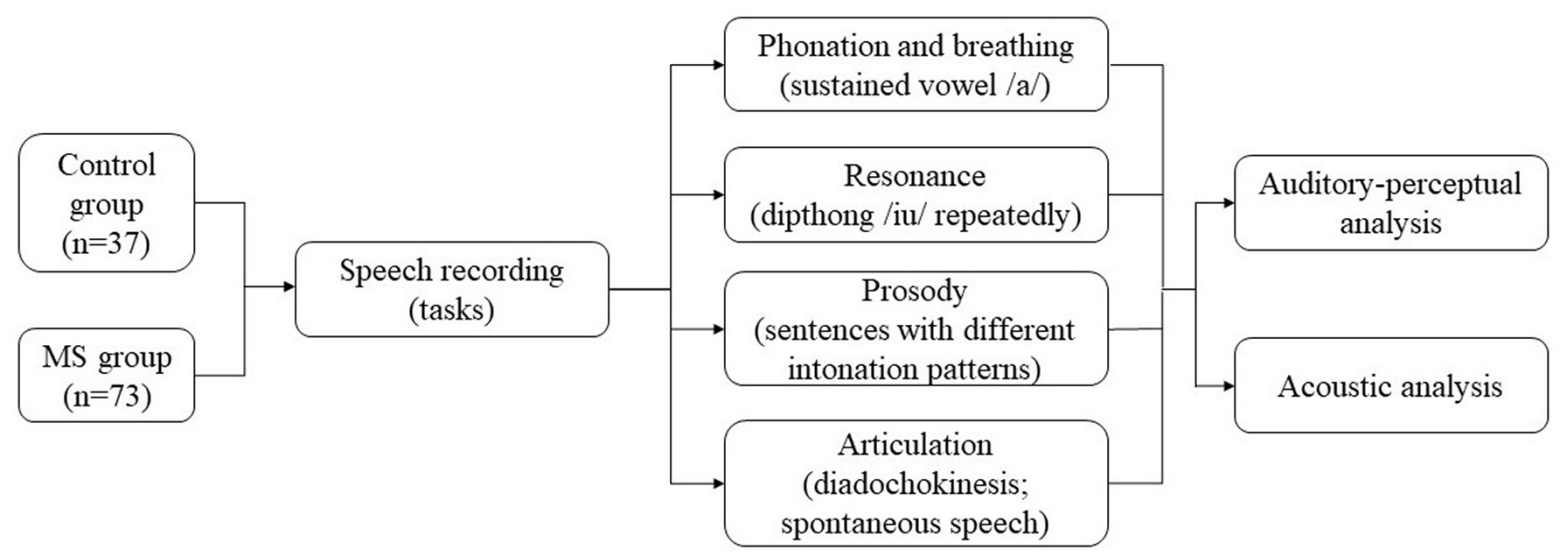
Experimental design. Developed by the author.

### 2.3. Speech assessment

#### 2.3.1. Data collection

Speech recording was performed using a TASCAM DR-07X speech recorder and a KARSECT HT-9 microphone positioned approximately 5 cm from the patient’s mouth, in a single session, in a quiet environment without acoustic isolation, both for patients and the group control. The recordings were sampled at 44.1 kHz and quantified at 16 bits. Both groups were asked to perform five tasks after a model provided by the researcher: (a) sustained vowel [a], in one breath, as long as possible; (b) repeated diphthong /iu/ in a single breath; (c) diadochokinesis /pataka/ as fast as possible in a single breath (19); (d) uttering the sentence “choveu muito neste fim de semana” (it rained a lot this weekend) in an affirmative, interrogative and exclamatory intonation; (e) spontaneous speech answering the question “what have you done today since you have been awake?” for 60 seconds.

#### 2.3.2. Auditory-perceptual analysis

The auditory-perceptual analysis (APA) (20, 21) is currently the gold standard in dysarthria assessment. Three trained speech therapists with at least 7 years of experience and blind to diagnosis conducted the APA with an agreement Kappa index ≥0.90. Before the speech analysis procedures, different speech alterations were presented and evaluated for auditory training. The examiners heard the speech samples in random order and analyzed the subsystems of speech (phonation, articulation, breathing, resonance, and prosody) based on the dimensions described by Duffy (22), classifying each one as (0) normal, (1) mild alteration, (2) moderate alterations, or (3) severe alterations. Afterward, the final diagnosis was indicated as (0) normal, (1) mild dysarthria, (2) moderate dysarthria, or (3) severe dysarthria.

#### 2.3.3. Acoustic analysis

Speech acoustic analysis (23) was performed in Praat (24) (version 6.1.55) with a script (25) to detect the intensity peaks automatically. As Brazilian Portuguese only allows for vowels at the syllable nucleus, counting the intensity peaks equals determining the number of syllables. This script’s reliability was evaluated by Jong and Wendel (2009) (25) comparing the analysis results manually made and performed with the PRAAT script used in this study. The analyzed parameters were based on Rusz et al. (2011) (26) and Vogel and Maruff (2008) (27). For phonation, the variables were Jitter (rap), Shimmer (local), Fundamental Frequency (F0 in Hz), the standard deviation of F0, and Harmonics-to-noise ratio (HNR), measured in a sustained vowel [a]. To characterize articulation, we used the following variables, obtained from the tasks of diadochokinesis (DDK) and spontaneous speech: number of syllables, number of pauses, total duration (in seconds), phonation time (total duration less pauses), phonation ratio (phonation time divided by total duration), speech rate (number of syllables divided by total duration), articulation rate (number of syllables divided by phonation time), average syllable duration (ASD), and the number of pauses weighted by total time. The ratio of the 2nd formant of the vowel [i] to the 2nd formant of the vowel [u] is a measure of vowel centralization, which indicates a reduced range of articulatory movements and is obtained from a sequence of repeated [iu]. Breathing capacity was estimated by the maximum phonation time (MPT). As for prosody, the F0 range was evaluated while producing a sentence with affirmative, interrogative, and exclamatory intonation. In Portuguese (other than, e.g., in German), the same sentence can be uttered as an affirmative or interrogative in the same word order by changing intonation. F0 range, i.e., the difference between F0 maximum and minimum values, indicates melodic variation and, thus, the speaker’s capacity to vary intonation (Figure 1).

### 2.4. Statistical analysis

Independent variables (age, sex, disease duration, age at onset of symptoms, EDSS, and disease subtype) and perceptual speech analysis were presented as descriptive analyses (absolute and relative frequencies and mean and standard deviation). Statistical tests were selected according to the distribution data provided by the Kolmogorov–Smirnov and Shapiro–Wilk tests. The Student’s T-test with Bootstrap was used for the acoustic analysis of the variables of the subsystems between the control group and the individuals with MS. Using Pearson’s correlation test with Bootstrap, correlations between independent and acoustic variables were performed. To compare the range of the variables in different groups, ANOVA was used with a *post hoc t*-test with Bonferroni correction. Statistical significance was defined at *p* < 0.05. The statistical software used was SPSS version 22.0.

The Bootstrap method makes it possible to obtain a 95% confidence interval for the parameter evaluated in each test and allows for the estimating parameters that make up a joint sample originating from the combination of individual samples from each population. It is possible to build an empirical distribution for the parameters regardless of the data distribution using the Bootstrap method (Efron & Tibshirani, 1998) (28). This observed distribution is assumed for the parameters and was used instead of the tabulated T distribution. Thus, there is no need for normality and data manipulation. All collected data were included in the analyses, and no outliers were excluded.

## 3. Results

The study included 73 MS patients and 37 healthy subjects in the control group. In the group of individuals with MS, 90.4% (*n* = 66) were non-smokers, 6.8% (*n* = 2) were former smokers, and 2.7% (*n* = 2) were active smokers. The control group was composed of 100% (*n* = 37) of non-smoking individuals. Table 1 shows the sociodemographic data of MS patients and the control group. The group of individuals with MS was matched with the group of controls by age and sex, with no statistically significant difference between them.

**Table 1:** Sociodemographic data.

	MS (*n* = 73)	Controls (*n* = 37)	value of *p*
	Mean (SD)	Mean (SD)	
Age (years)	47.04 (±11.74)	46.81 (±13.58)	0.927
Disease duration (years)	12.71 (±7.17)	-	
Age at onset of symptoms (years)	31.97 (±10.33)	-	
EDSS score	3.80 (±2.38)	-	

	*n* (%)	*n* (%)	
Sex female	50 (68.5%)	24 (64/9%)	0.830
Disease subtype			
RRMS	61 (83.6%)	-	
SPMS	9 (12.3%)	-	
PPMS	3 (4.1%)	-	

MS, Multiple Sclerosis; EDSS, Expanded Disability Status Scale; RRMS, relapsing–remitting multiple sclerosis; SPMS, secondary-progressive multiple sclerosis; PPMS, primary-progressive multiple sclerosis; SD, standard deviation.

Regarding the auditory perceptual analysis of speech (APA), 72.60% (*n* = 53) of MS patients were diagnosed with dysarthria. In the control group, 100% (*n* = 37) of the individuals were not dysarthric. Table 2 shows the percentage of normality and alteration of the patients with MS and the control group in the five speech subsystems.

**Table 2:** Auditory perceptual analysis—percent of normal and altered.

Speech subsystem	Classification	MS (*n* = 73)	Controls (*n* = 37)
Phonation	Normal	16 (21.9%)	33 (89.2%)
	Mild	48 (65.8%)	4 (10.8%)
	Moderate	8 (11.0%)	-
	Severe	3 (1.4%)	-
Breathing	Normal	32 (43.8%)	37 (100%)
	Mild	35 (47.9%)	-
	Moderate	6 (8.2%)	-
	Severe	-	-
Resonance	Normal	32 (43.8%)	35 (94.6%)
	Mild	35 (47.9%)	2 (5.4%)
	Moderate	6 (8.2%)	-
	Severe	-	-
Prosody	Normal	69 (94.5%)	37 (100%)
	Mild	2 (2.7%)	-
	Moderate	2 (2.7%)	-
	Severe	-	-
Articulation	Normal	42 (57.5%)	36 (97.3%)
	Mild	22 (30.1%)	1 (2.7%)
	Moderate	9 (12.3%)	-
	Severe	-	-

MS, Multiple Sclerosis.

Table 3 compares the acoustic analysis on the subsystem of phonation, breathing, and prosody between the control group and individuals with MS. There was a statistically significant difference in the variables F0 standard deviation measured on the sustained vowel/a/ (*p* = 0.001) and MPT (*p* = 0.001), even after redoing the analysis excluding the seven smokers from the sample of cases. There was also a significant difference in the frequency variation by sentence type. Table 4 compares the acoustic analysis on the subsystem of articulation (diadochokinesia, spontaneous speech and repeated diphthong/iu/in a single breath). As the PPMS group (*n* = 3) produced no pauses in the DDK task, these participants were excluded from the analysis comparing the results of pauses per second between the control and the MS participants. Table 5 shows the correlation between clinical disease data (age at onset of symptoms, disease duration, and EDSS score) and altered speech acoustic variables. A regression analysis confirmed that the EDSS results might be relevant to the phonation time at the spontaneous speech task (*r*^2^ = 0.056, adjusted *r*^2^ = 0.043; Anova: *F* = 4.247, *p* = 0.043) and the decreased functionality but only among the most severe subtypes of the disease.

**Table 3:** Comparison of acoustic analysis measures between the control and MS groups in the speech subsystem of phonation, breathing and prosody.

Speech subsystem	Variables	MS (*n* = 73)	Controls (*n* = 37)	value of *p*
Phonation	F0 mean (Hz)	175.76 (±48.71)	180.42 (±51.38)	0.610
	F0 standard deviation (Hz)	15.79 (±19.46)	3.20 (±6.54)	0.001*
	HNR (dB)	21.83 (±4.78)	20.73 (±6.04)	0.312
Breathing	MPT (sec)	11.09 (±6.79)	17.80 (±7.39)	0.001*
Prosody				
Affirmative sentence	Frequency variation (Hz)	344.32 (±223.50)	432.62 (±204.30)	0.041*
	Intensity variation (dB)	32.68 (±6.92)	31.74 (±8.01)	0.531
Exclamatory sentence	Frequency variation (Hz)	370.71 (±209.68)	430.49 (±196.23)	0.144
	Intensity variation (dB)	33.54 (±6.44)	33.75 (±7.92)	0.900
Interrogative sentence	Frequency variation (Hz)	369.99 (±227.96)	443.41 (±192.74)	0.077
	Intensity variation (dB)	32.56 (±6.66)	30.24 (±6.55)	0.089

MS, Multiple Sclerosis; F0, fundamental frequency; HNR, harmonics-to-noise ratio; MPT, maximum phonation time; Db, decibels; data are presented as mean and standard deviation; *p < 0.05.

**Table 4:** Comparison of acoustic analysis measures between the control and MS groups at the articulatory subsystem.

Evaluated task	Variables	MS (*n* = 73)	Controls (*n* = 37)	Value of *p*
dipthong /iu/repeatedly	F2i/F2u	2.49 (±0.63)	2.52 (±0.38)	0.803
Diadochokinesis/pataka/	Number of syllables	48.56 (±31.20)	62.01 (±25.71)	0.020*
	Duration	9.68 (±5.20)	12.14 (±5.01)	0.022*
	Phonation time	9.30 (±5.16)	11.99 (±5.04)	0.011*
	Phonation ratio	96.21% (±8.64%)	98.64% (±4.02%)	0.060
	Speech rate	4.92 (±1.45)	5.15 (±1.32)	0.386
	Articulation rate	5.07 (±1.40)	5.27 (±1.21)	0.424
	ASD	0.23 (±0.14)	0.20 (±0.05)	0.165
	Pauses/s. (*n* = 107)	0.072 (±0.13)	0.027 (±0.09)	0.031*
Spontaneous speech	Number of syllables	185.22 (±46.38)	201.11 (±54.71)	0.147
	Duration	58.57 (±6.88)	57.64 (±8.52)	0.596
	Phonation time	43.25 (±9.24)	47.25 (±9.98)	0.043*
	Phonation ratio	74.30% (±14.18)	82.05% (±12.04%)	0.003*
	Speech rate	3.18 (±0.73)	3.48 (±0.78)	0.054
	Articulation rate	4.26 (±0.43)	4.19 (±0.72)	0.605
	ASD	0.23 (±0.02)	0.24 (±0.03)	0.507
	Pauses/s. (*n* = 110)	0.27 (±0.1)	0.23 (±0.12)	0.061

MS, Multiple Sclerosis; F2i/F2u, ratio of the 2nd formant of the vowel /i/to the 2nd formant of the vowel/u/; ASD, average syllable duration; *p < 0.05. As the PPMS group (*n* = 3) produced no pauses in the DDK task, these participants were excluded from the analysis comparing the results of pauses per second between the control and the MS participants.

**Table 5:** Correlation between clinical data and altered acoustic variables—MS group.

	Age at onset of symptoms		Disease duration		EDSS	
	Value of *p*	*r*	Value of *p*	*r*	Value of *p*	*r*
F0 standard deviation (Hz)	0.034*	0.249	0.095	-	0.087	-
MPT (sec)	0.474	-	0.918	-	0.740	-
Affirmative sentence frequency variation (Hz)	0.424	-	0.870	-	0.162	-
Diadochokinesis						
No. of syllables	0.601	-	0.138	-	0.344	-
Duration	0.992	-	0.651	-	0.693	-
Phonation time	0.936	-	0.695	-	0.708	-
Pauses/s.	0.098	-	0.426	-	0.098	-
Spontaneous speech						
Phonation time	0.203	-	0.359	-	0.043*	−0.238
Phonation ratio	0.159	-	0.150	-	0.023*	−0.265

EDSS, Expanded Disability Status Scale; MPT, maximum phonation time; *p < 0.05.

## 4. Discussion

This study found a higher prevalence of dysarthria in individuals with MS in the APA than previously reported (13, 14, 16), with 72.6% (*n* = 53) of this sample characterized as dysarthric. Regarding the performance of patients with MS in the subsystems of speech individually, 78.2% (*n* = 57) presented alterations in phonation, 56.8% (*n* = 41) in breathing, 56.8% (*n* = 41) in resonance, and 42.4% in articulation.

As in most studies (13, 29, 30), in the acoustic assessment of speech, individuals with MS showed a more significant variation of F0 during the emission of the sustained vowel/a/as compared to the control group, evidenced by the values of the F0 sd. However, the difference in mean F0 between these two groups was not significant. These data indicate that this frequency variance is due to pitch breaks (9, 31). Jitter is defined as the parameter of frequency variation from cycle to cycle. Local Jitter is the average absolute difference between consecutive periods divided by the average period (24). The limit of normality of jitter value according to Praat (24) is up to 1.040%. The mean jitter value in patients with MS was 0.447% (±0,551), within the normal range. Shimmer is defined as the parameter of the amplitude variability of the sound wave from cycle to cycle. Shimmer offers an indirect perception of noise in vocal production, and its values increase the greater the amount of noise in an emission. The value of the standard of normality according to Praat (24) is up to 3.81%. The mean value of shimmer in patients with MS was 2.493% (±2,299), within the normal range.

An important finding was the significantly lower value of MPT in patients with MS, indicating an alteration in breathing or the coordination of breathing with speech, which may show cerebellar alteration. Nordio (16) (2018) evaluated and found a significant reduction in the maximum expiration time, a variable that negatively correlated with the EDSS score. The maximum expiration time correlated positively with the maximum phonation time. This reduction in MPT may be associated with fatigue since it is estimated that 75 to 96% of MS patients have these symptoms (32). The differences in MPT and F0 SD could be influenced by the presence of smokers in the group of patients. Thus, the analysis was performed again, excluding the group of smokers. There was no change in the results, and these variables remained significant. Therefore, it is possible to observe that these alterations are not associated with smoking in this sample but with the pathophysiology of MS itself.

Unlike the findings in previous studies, which described prosody as one of the subsystems with the largest alteration (7, 9, 13, 33), in this sample, no significance was found in this data since only 5.4% (*n* = 4) of the patients showed changes in the APA. However, individuals with MS showed a decrease in the frequency variation in the enunciation of the affirmation sentence compared to the control group, a possible indication of difficulty in uttering sentences that require less variability while maintaining the ability to perform more significant variation in exclamatory and interrogative sentences. Therefore, there is a change in the patients’ expressiveness. It is important to note that the participant was asked to repeat the phrase in an affirmative tone during the evaluation, and no sentence reading was requested, which could explain less frequency variation.

Another interesting result was that, although patients with MS had an adequate speech rate in the diadochokinesis and spontaneous speech tasks (they showed no changes in F2i/F2u and ASD), during diadochokinesia, they differed from the control group in duration time, the number of syllables, the number of pauses per seconds and the time of phonation. Thus, patients with MS presented irregular diadochokinesia with pauses during the task, although within the same breath, suggestive of changes unrelated to the decreased range of motion of orofacial structures involved in speech production, which usually occurs in other pathologies to compensate for the reduced speech rate (11, 12).

In spontaneous speech, the phonation time and ratio altered, with increased pauses, in line with Noffs et al. (2018) (8) findings. The speech rate, including pause times, informed the number of syllables per second, and presented a value close to significance (*p* = 0.054). In the articulation rate, which is the number of syllables per second without the time of pauses, the patients’ results were even minimally higher than that of the control group. These findings indicate that the difficulty of these subjects is not entirely of articulatory origin but is somehow impaired by a lack of coordination among breathing, phonation, and articulation since these alterations impair articulatory variables. Spontaneous speech could also measure language disorders. So, phonation time in spontaneous speech can also be related to their will or ability to speak, which are reasons that are hard to distinguish.

Some MS patients show alterations of cerebellar origin and may have difficulties programming a sequence of movements before its onset (34), a factor that may contribute to irregular syllabic sequencing (9), causing an increase in the number of pauses during speech. These data support Hartelius et al. (2000) (35) hypothesis, which classifies temporal deregulation as a regular feature in dysarthria in MS. In future studies, it would be interesting to relate these findings to the EDSS-specific cerebellar, pyramidal, and brainstem functional assessments proposed by Rusz (2018) (9). This data is relevant since a negative, albeit weak, correlation was found between the phonation time, the phonation ratio, and the EDSS score. Therefore, the more pauses in speech, the higher the EDSS score. Cognition alterations or fatigue could influence the increased number of pauses.

A factor that has yet to be analyzed and described in the literature regarding dysarthria in individuals with MS is resonance. Only two studies mentioned the occurrence of hypernasal voice (7, 30). More than 50% of the patients in this study had hypernasality verified in APA, reflecting changes in this subsystem. Inadequate nasality is associated with insufficient velopharyngeal closure (36). There is significant variability in age among the MS patients in the sample, with a minimum age of 20 years and a maximum of 73 years. Therefore, the results of this study could be attributed to non-disease-related differences between the subjects, such as age, since one founds changes in speech associated with aging also among healthy individuals. Noffs et al. (2020) (30) report the results of an unpublished study that compared the variability of speech among healthy subjects with individuals with MS at different stages of the disease, with changes being observed in a significantly greater number in the group with MS than among the controls. In our sample, there were no age-related speech alterations in the control group since they were matched by sex and age.

In the regression analysis of the significantly correlated variables, EDSS best explains the decrease in phonation time in individuals with MS, but with a very small R^2^, indicating other factors not investigated here. Regression analysis shows that the older the individual, the lower the phonation ratio during spontaneous speech. At the same time, with each level that the EDSS score increases, there is a decrease in the phonation ratio. Thus, it seems that the contribution of the EDSS in this analysis is more important than the contribution of age. As others have already affirmed, the longer the disease duration, the higher the EDSS score, other things being equal. Disease subtype, the number of relapses, the number of lesions on MRI, the presence of Gadolinium-enhancing lesions on MRI, how previous relapses were managed, and which MS medication patients are currently using, among other factors, could influence that relationship.

A regression analysis ruled out the hypothesis of a random positive correlation between F0 sd and age at the onset of symptoms, and, contrary to expected, the longer the disease duration, the lower the F0 sd. As most of our MS patients belong to the RRMS subtype, they present with a long time of disease and a lower EDSS score since this subtype represents the least aggressive disease phenotype in comparison with PPMS and SPMS, which would justify this finding.

There was no significance when performing the correlation analysis between clinical data and speech variables only with individuals in the RRMS group, indicating that the EDSS can help predict the decrease in speech functionality in the most severe manifestations of MS. However, it cannot be used in a general way. We found individuals with a mild clinical disability (EDSS<2.0) who have dysarthria, as well as non-dysarthric individuals with a more severe disability (EDSS>4.0). Therefore, other factors besides disability, as reflected in EDSS, play a role in the manifestation of dysarthria, such as the presence of brainstem and cerebellar lesions. No significant correlation was observed between speech variables and clinical data regarding the disease duration or age at the onset of symptoms. Speech alterations do not necessarily go hand in hand with motor alterations, so studies should aim to analyze the speech pattern and compare it with the clinical aspects of the disease.

A limitation of this study was the small number of patients with the PPMS and SPMS forms, making it impossible to compare speech data with the RRMS group. Considering MS natural history, this difference in proportion between the groups is expected. Furthermore, our data were collected transversal and consecutive at the neuroimmunology outpatient clinic, which resulted in a proportion like that expected from the literature (37). Future longitudinal studies are needed to understand better the evolution of speech patterns presented by MS patients and factors that are interrelated with these changes. Other limitations were the cross-sectional design, that does not prove causation; no previous literature evaluating MS patients with the same Praat script in Brazilian Portuguese. The acoustic measurements in this study were those used in speech therapy clinical practice.

## 5. Conclusion

The speech profile found in MS patients in this sample was mild dysarthria, with changes in the speech subsystem of phonation, breathing, resonance, and articulation, respectively, in order of prevalence. The auditory-perceptual and acoustic speech analysis findings were corresponding and complementary, although the acoustic analysis was more sensitive to verifying lack of coordination among respiration, phonation and articulation, and speech functionality. It reinforces the importance of using both methods in parallel to assess and better understand speech disorders.

A correlation was found between the high number of pauses performed during spontaneous speech and the EDSS score, indicating that the severity of the disease may be associated with lower phonation ratio in MS. No significant correlation was found between speech variables and clinical data on disease duration or age at onset of symptoms. Speech analysis demonstrates the potential to aid in the diagnosis, progression monitoring, and treatment of MS and has rapid and practical clinical applicability.

## Data availability statement

The original contributions presented in the study are included in the article/supplementary material, further inquiries can be directed to the corresponding author.

## Ethics statement

The studies involving human participants were reviewed and approved by CPA Ethics Committee under No. GPPG 2019-0789. The patients/participants provided their written informed consent to participate in this study.

## Author contributions

MK, AF, RR-N, and MO: research design. MK, VK, VS, AA, and IK: recruitment of patients and data collection. MK, VS, AA, RR-N, and MO: tabulation, statistical analysis, tables, and figures. MK, AF, VK, VS, AA, RR-N, and MO: writing. MK, VK, VS, AA, RR-N, and MO: text review and addition of significant parts. All authors contributed to the article and approved the submitted version.

## Funding

This work was supported by the Hospital de Clínicas de Porto Alegre - FIPE (GPPG-HCPA 2019-0789) and Rothe-Neves, R was supported by Conselho Nacional de Desenvolvimento Científico e Tecnológico - Brasil (nº 316036/2021-8).

## Conflict of interest

The authors declare that the research was conducted in the absence of any commercial or financial relationships that could be construed as a potential conflict of interest.

## Publisher’s note

All claims expressed in this article are solely those of the authors and do not necessarily represent those of their affiliated organizations, or those of the publisher, the editors and the reviewers. Any product that may be evaluated in this article, or claim that may be made by its manufacturer, is not guaranteed or endorsed by the publisher.
